# Mitigating citrus fruit cracking: the efficacy of chelated calcium or silicon foliar fertilizers in ‘Okitsu no. 58’ citrus fruit

**DOI:** 10.3389/fpls.2024.1402945

**Published:** 2024-07-24

**Authors:** Tie Wang, Liping Tan, Zhaofang Chen, Youting Yang, Ya Yuan, Zhendong Zheng, Lijun Deng, Mingfei Zhang, Guochao Sun, Siya He, Jun Wang, Bo Xiong, Zhihui Wang

**Affiliations:** ^1^ College of Horticulture, Sichuan Agricultural University, Chengdu, China; ^2^ The Industrial Crop Institute, Dazhou Academy of Agricultural Sciences, Dazhou, China

**Keywords:** foliar fertilizer, mineral elements, pectin, enzyme activity, fruit quality

## Abstract

The ‘Okitsu No. 58’ citrus variety is highly prone to fruit cracking, which jeopardizes yield and results in economic losses. In this study, we investigated the impacts of spraying 5 distinct concentrations (0.1, 0.2, 0.3, 0.4, and 0.5 g/L) of chelated calcium (Ca) or silicon (Si) fertilizers at the young fruit stage (60-90 days after flowering, DAF) on fruit cracking and quality in the citrus variety ‘Okitsu No. 58’. The results showed either Ca or Si fertilizer treatments reduced fruit cracking. We found that all Ca and partial Si treatments (0.4 and 0.5 g/L) significantly promoted the accumulation of Ca content in the peel. Notably, Ca or Si treatments significantly reduced polygalacturonase (PG) activity and inhibited the production of water-soluble pectin (WSP) in the peel. Additionally, Ca or Si treatments elevated the superoxide dismutase (SOD) activity and decreased the malondialdehyde (MDA) content of the peels. Changes in these parameters likely contributed to strengthening the durability of peel cell wall constituents, thus enhancing the fruit’s resistance to fruit cracking. Overall, except for the C3 (0.3 g/L of Ca), Ca or Si fertilizers contributed to fruit conventional quality, mainly in terms of higher soluble sugars (SS) and SS/TA (titratable acid). Therefore, our findings will provide a reference for the prevention and control of citrus fruit cracking and the development of new fertilizers.

## 1 Introduction

Citrus is one of the most popular horticultural crops in the world (Wang et al., 2020). Citrus fruits are consumed in large quantities because of their attractive aroma and flavor as well as their rich nutritional and biological activities, which provide high nutritional value and various health-promoting effects (Lu et al., 2021; Sun et al., 2023). However, the fruits of some cultivars are prone to fruit cracking, leading to yield loss and significant commercial losses to growers (Li and Chen, 2017; Huang et al., 2024). ‘Okitsu No. 58’ (*Citrus reticulata* Blanco, (Sweets pring tangelo × Terovita orange) × [*Citrus reticulate* ×(*C.reticulata×Csinenesis*)]) is a high-quality late maturing hybrid citrus variety introduced to China in recent years. Its pulp has high sugar content and has important market value and market competitiveness (Zhai et al., 2022). However, according to our investigation, ‘Okitsu No. 58’ has a serious fruit-cracking problem during the fruit expansion stage, which significantly inhibits the sustainable development of this cultivar (Huang et al., 2024).

According to research, both internal and external conditions can underly the dynamics of fruit cracking (Fischer and Álvarez-Herrera, 2021). Intrinsic factors mainly include the genetic characteristics of the variety (Li et al., 2009) and rootstock variety characteristics (Agustí et al., 2003), whereas the extrinsic environment mainly includes environmental conditions and cultivation management levels (Measham et al., 2009). It is well known that plant growth and development are related to numerous factors, but the availability of adequate nutrients is the main focus (Davarpanah et al., 2018). Previous studies show that nutritional deficits in some peels can cause them exhibiting metabolic and developmental issues, further exacerbating fruit wrinkling and cracking (Li and Chen, 2017). Calcium (Ca) is an important mineral nutrient involved in citrus fruit development (Storey and Treeby, 2002; Dong et al., 2009; Tam et al., 2012). Previous studies in ‘Hongjiang’ orange citrus found that foliar spraying of Ca nitrate during rapid fruit expansion could increase Ca content in citrus peel and further inhibit the expression of cell wall degrading enzyme genes in the peel, thus reducing the occurrence of fruit cracking (Huai et al., 2022). In addition, it was also found that Ca treatment significantly increased the expression of antioxidant-related genes in the citrus peel, improved antioxidant enzyme activity, and maintained the balance of reactive oxygen species metabolism, thus reducing fruit cracking (Zhang et al., 2021). To further determine how Ca reduces susceptibility to fruit cracking Schumann et al., (2022), studied the effects of Ca on the cell wall under a microscope, they found that Ca reduces swelling, the susceptibility of the fruits to cracking. Besides the focus on Ca in fruit cracking, silicon (Si) also been emphasized and applied for fruit cracking control (Landi et al., 2016). Si is one of the components of plant cell walls; it increases the strength of plant stalks, and improves the resistance to stress (Qin et al., 2016). In addition, Si has the ability to strengthen the cell walls of fruit tree plants, improving plant health and productivity (Etesami and Jeong, 2018) Lü et al., (2021). demonstrated that proper Si fertilizer treatment significantly reduced the occurrence of watermelon fruit dehiscence. However, the application of Si fertilizer in fruit trees, especially for fruit cracking, has been reported relatively little. Overall, the research on Si nutrition in fruit trees is still in its infancy, and the discovery of related mechanisms needs to be further developed (Wang et al., 2021). Hence, we speculate that optimal concentrations of Ca and Si fertilizers exert a suppressive effect on fruit cracking in ‘Okitsu No. 58’ citrus. However, the extent of their effectiveness remains unreported.

Fruit cracking is closely related to peel cell wall substances, hydrolytic enzymes, and antioxidant enzymes (Guo et al, 2019; Choi et al., 2020). Our previous investigation into cell wall composition in crack-prone and non-crack-prone citrus varieties revealed that the morphological transformation of pectin and the degradation of hemicellulose and lignin in citrus peel are significant contributors to fruit cracking in crack-prone citrus varieties (Huang et al., 2024). Among the enzyme activities affecting fruit cracking, researchers studying navel oranges found that cell wall hydrolases can weaken peel strength by breaking down cellular polysaccharides. This was evidenced by the increased activities of pectinase, pectin methyl esterase, xylanase, and peroxidase in the peel (Li, 2009; Li and Chen, 2017). Additionally, fruit cracking is closely associated with antioxidant enzyme activities. For instance Zhang et al., (2021), conducted a study where citrus plants were treated with Ca fertilizer spraying. They observed that Ca ions notably upregulated the expression levels of *CsSOD*, *CsCAT*, *CsAPX*, and *CsGPX*, while downregulating the expression level of *CsPOD*. This led to heightened activities of SOD, CAT, APX, and GPX, and a reduction in POD activity. These alterations in enzyme activities resulted in enhanced peel cell wall integrity and delayed fruit cracking onset. Studies on tomatoes have found that cracked fruit had significantly lower SOD levels compared to normal fruits, possibly due to inactivation of the enzyme by reactive oxygen species (ROS) or reduced enzyme synthesis (Zhang et al., 2020). POD is a common oxidoreductase in plants that can cross−link phenolic groups of cell wall constituents, which leads to a decrease in peel extensibility (Elstner, 1982; Campa, 1991).

Current research has established the definitive impact of Ca on citrus fruit quality. For instance, in Newhall navel orange trees, foliar application of Ca(NO_3_)_2_ during the physiological fruit drop stage not only significantly increases individual fruit weight but also affects the metabolism of fruit titratable acidity (TA), thereby enhancing fruit maturity (Zheng et al., 2017). In studies on Tarocco oranges, it was found that with the increase in Ca fertilizer concentration, the levels of total soluble solids (TSS), total sugars, and the ratio of TSS to TA generally decreased, while fruit TA levels tended to increase (Wang et al., 2012). Furthermore, a composite product of Ca and Si fertilizer applied via foliar spray before harvest also plays a role in regulating citrus fruit quality. Specifically, it delays fruit ripening, increases TSS, TA, ascorbic acid, total phenolics, and total antioxidant capacity, while reducing fruit decay during shelf storage (Ziogas et al., 2022). Although no specific studies on the impact of sole Si fertilizer application on citrus fruit quality have been documented, such effects have been reported in strawberries (Dou et al., 2023; Xu et al., 2023). Researchers found that foliar spraying of Si on leaves helps increase strawberry yield and quality. Based on the aforementioned studies, we speculate that Si also significantly influences citrus fruit quality.

In summary, both Ca or Si fertilizer treatments demonstrated significant effects on fruit cracking and quality across different varieties, albeit with varying treatment concentrations among them. To identify suitable fertilization concentrations for ‘Okitsu No. 58’ citrus, we established different concentration gradients of Ca or Si fertilizer treatments in this experiment. Subsequently, we examined their combined effects on fruit cracking and overall fruit quality of ‘Okitsu No. 58’ citrus. The findings from this investigation will serve as a valuable reference for controlling citrus fruit cracking.

## 2 Materials and methods

### 2.1 Plant materials and experimental design

In this experiment, high grafted 3-year-old ‘Okitsu No. 58’ citrus was used as test material. Trees were grafted on ‘Newhall navel orange’ (*Citrus sinensis* Osbeck cv. Newhall) interstock with ‘red tangerines’ (*Citrus reticulata* Blanco) as the rootstock. The spacing between plants and rows of all trees is 3×5 m, which were managed using standard horticultural practices as reported (Chen et al., 2019). The test site was located in Linqiong Town, Qionglai City, Chengdu City, Sichuan Province, China, at 103°09′E, 30°33′N, with an elevation of 504 m. The region has a subtropical monsoonal and humid climate. The average annual temperature was 16.9°C, the total annual sunshine was 1107.9 h, the average annual rainfall was 1117.3 mm, and the annual frost−free period is 330 d.

Ninety−nine citrus plants of ‘Okitsu No. 58’ with uniform growth and close proximity were randomly selected in the experimental field, and 3 plants were set as one treatment with 3 replications. Each treatment was evenly distributed among the three rows. Chelated Ca fertilizer (Ca = 30 g·L^−1^, amino acid = 100 g·L^−1^) (Sichuan Runer Technology CO.,LTD, China), and Si fertilizer (SiO_2_ = 290 g·L^−1^, nano-powder silicon source, particle size 8-10 nm, pH=5.0) (Chengdu Huahong Biotechnology Co., China) were used as fertilizers to provide Ca and Si elements. It is worth noting that the amino acid inside chelated Ca fertilizer is more of a chelating agent, and have a limited impact on fruit cracking (Zhao et al., 1995; Mohamed et al., 2020).

Five gradient concentrations of 0.1, 0.2, 0.3, 0.4, and 0.5 g·L^−1^ were set for Ca and Si fertilizers according to fertilizer use recommendations and reference to previous studies (Chen et al., 2014; Landi et al., 2016). The corresponding dilutions were 300, 150, 100, 75 and 60 for Ca fertilizer and 2900, 1450, 966.70, 725 and 580 for Si fertilizer. The measured fertilizer is meticulously mixed with the appropriate volume of water and subsequently foliar-sprayed at 60, 75, and 90 days after flowering (DAF) until excess water drips from both the foliage and fruit surface. Importantly, Ca or Si foliar fertilizers are administered separately. In the experiment, water served as a control check (CK), with specific treatments outlined in **Table 1**.

**Table 1:** The concentration and timing of Ca and Si fertilizer applications.

Treatment	Application concentration (g/L)		
	60 d	75 d	90 d
C1	Ca 0.1	Ca 0.1	Ca 0.1
C2	Ca 0.2	Ca 0.2	Ca 0.2
C3	Ca 0.3	Ca 0.3	Ca 0.3
C4	Ca 0.4	Ca 0.4	Ca 0.4
C5	Ca 0.5	Ca 0.5	Ca 0.5
S1	Si 0.1	Si 0.1	Si 0.1
S2	Si 0.2	Si 0.2	Si 0.2
S3	Si 0.3	Si 0.3	Si 0.3
S4	Si 0.4	Si 0.4	Si 0.4
S5	Si 0.5	Si 0.5	Si 0.5
CK	water	water	water

C, Ca treatment; S, Si treatment; CK, control check.

### 2.2 Sample collection and processing

Sampling started from 110 DAF, and the sampling time was 110, 130, 150, 170, 190, 210, and 280 DAF. A total of 10 fruits of medium size, free from diseases and pests, and mechanical damage, were randomly selected from the upper and middle layers of the outer canopy of each tree. The fruits were brought back to the laboratory in ice boxes for processing, washed, and the peel and flesh were separated. Part of the peel was snap−frozen in liquid nitrogen and stored in a −80°C refrigerator to determine relevant enzyme activities. The other part of the sample was dried to constant weight in an oven at 60°C and stored in a dehumidifier for determination of cell wall material, mineral elements, etc.

### 2.3 Fruit cracking rate

The percentage of cracked fruits was counted at 20−day intervals from the appearance of the cracked fruits. Fruit cracking rate = number of cracked fruits per plant/total number of fruits per plant (Different developmental periods); values are shown as percentages (%).

### 2.4 Mineral elements

A dry sample of 200 mg of crushed peel was carbonized in an electric furnace and then placed in a high−temperature resistance furnace at 550°C for 6 h. After cooling, the sample was dissolved in 2 mL of concentrated hydrochloric acid and fixed to 100 mL with distilled water, and the Ca or Si contents were determined using the Shimadzu AA6300G atomic absorption spectrophotometry (Hamamatsu Photonics, Hamamatsu, Japan) (Mohammadzai et al., 2010), results were expressed in g/Kg DW.

### 2.5 Cell wall polysaccharides

The dried sample (2 g) was accurately added to 100 mL of 80% (v/v) ethanol, boiled in a water bath for 20 min, cooled, centrifuged at 3000 × g for 10 min, and the residue was thoroughly washed three times with 15 mL of 80% (v/v) ethanol. The filter residue was collected, and the water−soluble pectin (WSP), CDTA−soluble pectin (CSP), Na_2_CO_3_−soluble pectin (NSP), hemicellulose (HC), and cellulose (CEL) contents were determined according to previously reported methods (Wang et al., 2015). The pectin content in the fraction was determined by the m−hydroxydiphenyl method using galacturonic acid (GA) as a standard and was expressed as mg g^−1^FW. WSP, CSP, and NSP values were summed to obtain the total pectin (TP) content. The hemicellulose and cellulose fractions were estimated to be glucose using the anthrone method. The results were expressed as mg g^−1^ DW.

### 2.6 Cell wall enzyme activity, antioxidant enzymes, and malondialdehyde (MDA)

Determination of PG activity: 2 g of the sample to be tested was taken, 95% ethanol was used for enzyme extraction, and then the absorbance was measured at 540 nm with reference to the previous measurement method (Krebbers et al., 2003). The PME activity was determined by extracting 1 g of the sample to be tested with 2 mL of 5% NaCl solution in an ice bath and following the previous method of treatment (Krebbers et al., 2003). The endoglucanase (EG) activity was measured using the DNS method (Andrews and Li, 1995). SOD and POD activities were measured as previously described (Liao et al., 2015). All enzyme activity units were U/g FW. MDA content was measured using the method described by Bian et al (Bian et al., 2018), the results were expressed as μmoL/g FW.

### 2.7 Fruit quality

Single fruit weights were weighed using an electronic balance (AL204, METTLER, Switzerland). The total soluble solid (TSS) and titratable acid (TA) contents were determined using an integrated sugar and acid machine (Pocket PAL−BXIACID1, ATAGO, Japan), with results expressed as a percentage (%). Soluble sugars (SS) were determined using the anthrone colorimetric method (Bian et al., 2018). Accurately weigh 0.25 g of crushed fruit pulp sample, place it in a 15 mL centrifuge tube, add 10 mL of deionized water, immerse it in a boiling water bath for 1 hour, let it cool naturally, and then centrifuge it at 8000 rpm for 3 minutes. The supernatant was then used for the determination of soluble sugar. The content of vitamin C (Vc) was determined by spectrophotometry (Kelebek and Selli, 2014), with units expressed as mg/100mL. Accurately aspirate 5 mL of the juice, dilute it with 1% oxalic acid, and bring the volume to 50 mL. Pipette another 5 mL of the diluted solution into a 50 mL triangular flask, and add 2, 6-dichlorophenolindophenol through a semi-microburette until the solution turns pink in color and remainsstable for 30 seconds. Record the amount of 2, 6-dichlorophenolindophenol consumed, and calculate the Vc content.

### 2.8 Statistical analysis

Statistical analysis was performed via the use of the IBM SPSS Statistics 23.0 (IBM, Armonk, NY, USA), with the use of a one-way analysis of variance (One-way ANOVA) (Tukey’s test; different letters in the figures indicate differences for *P*<0.05). Principal component analysis (PCA) at https://www.omicstudio.cn/tool/13. The correlation Network was constructed using OmicStudio tools at https://www.omicstudio.cn/tool.

## 3 Results

### 3.1 Fruit cracking rate

**Figure 1:**
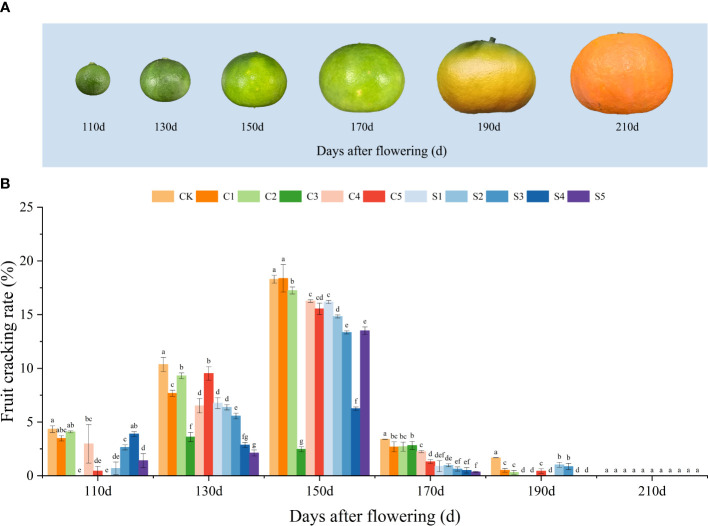
Developmental status of ‘Okitsu No. 58’ citrus fruits **(A)** and the effect of different treatments on fruit cracking rate **(B)**. C, Ca treatment; S, Si treatment. The parameter values presented in each figure are indicated as mean ± standard error (n=3). Different letters denote statistically differences between different treatments (Tukey test, *P* < 0.05).

The growth status and fruit cracking rate of ‘Okitsu No. 58’ citrus at different developmental stages are depicted in **Figure 1**. Through extensive observation, it was determined that fruit cracking commenced at 110 DAF, reached its peak at 150 DAF, and ceased by 190 DAF. Across each developmental phase, the CK exhibited a notably higher crack rate, particularly evident at 150 DAF, where the cracking rate soared to 18.29%. In contrast, the Ca or Si treatments demonstrated significantly lower cracking rates, suggesting that either Ca or Si fertilizers suppressed fruit cracking, with a more pronounced effect observed in C3 and S4 treatments. In the case of silicon fertilizer application, except for 110 DAF and S5 of 150 DAF, the other treatments showed a decreasing trend in fruit cracking with increasing Si fertilizer concentration.

### 3.2 Mineral elements

**Figure 2:**
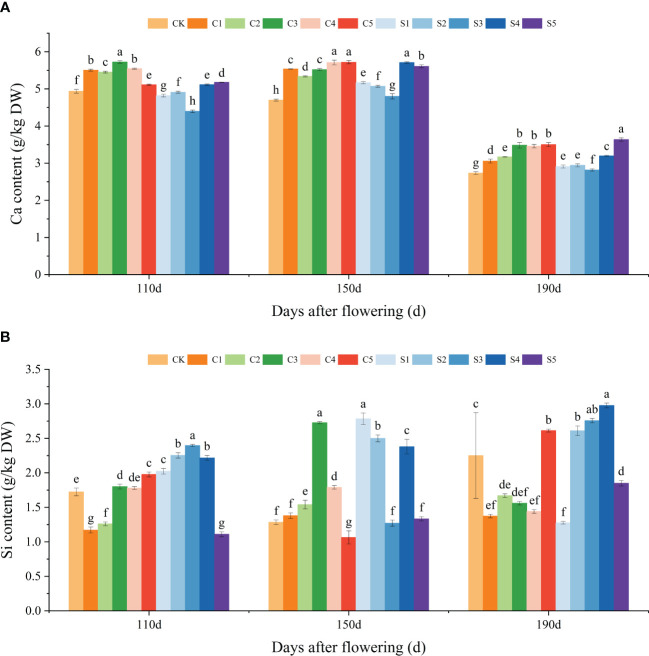
Effect of different treatments on the elemental Ca and Si content of the ‘Okitsu No.58’ citrus peel. **(A)** Ca, **(B)** Si. C, Ca treatment; S, Si treatment. The parameter values presented in each figure are indicated as mean ± standard error (n=3). Different letters denote statistically differences between different treatments (Tukey test, *P* < 0.05).

Aligned with the fruit cracking rate (**Figure 1B**), ‘Okitsu No. 58’ citrus fruits at 110, 150, and 190 DAF were chosen for the assessment of Ca and Si accumulation and subsequent in-depth analysis. Our results showed that Ca treatments significantly increased Ca accumulation in the peel, especially in the C3, C4 and C5 treatments, whereas among the Si treatments S1, S2 and S3 treatments had no significant effect on Ca content. However, both S4 and S5 treatments significantly facilitated peel Ca accumulation (**Figure 2A**). Particularly noteworthy is the observation that at 190 DAF, the peel Ca content of S5 was significantly highest, measuring at 3.64 g/kg DW. Moreover, the C3 treatment at 110 DAF exhibited the highest peel Ca content, registering at a significant level of 5.72 g/kg DW. Regarding peel Si content, we observed that C3 demonstrated a promotional effect on Si from 110 to 150 DAF, but this trend reversed at 190 DAF. Additionally, S2 and S4 treatments promoted Si enrichment in the fruit peel, whereas all S5 treatments exhibited inhibition, possibly due to high Si concentrations causing polymerization reactions thereby reducing uptake by citrus leaves (**Figure 2B**). In summary, C3, C4, C5, S4, and S5 treatments notably enhanced Ca enrichment in the peel, whereas only S2 and S4 facilitated Si accumulation. Furthermore, the majority of Ca treatments exhibited a greater promotion of peel Ca enrichment compared to Si treatments, while Si treatments induced a higher degree of Si enrichment than Ca treatments.

### 3.3 Cell wall polysaccharides

**Figure 3:**
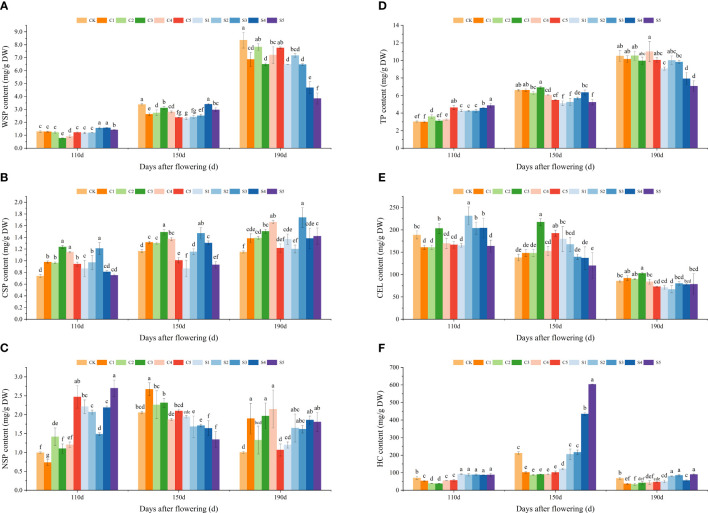
Effect of different treatments on the polysaccharide content of the cell wall of the ‘Okitsu No.58’ citrus fruit peel. **(A)** WSP, **(B)** CSP, **(C)** NSP, **(D)** TP, **(E)** CEL, **(F)** HC. C, Ca treatment; S, Si treatment. The parameter values presented in each figure are indicated as mean ± standard error (n=3). Different letters denote statistically differences between different treatments (Tukey test, *P* < 0.05).

The effects of various treatments on the cell wall polysaccharide contents of ‘Okitsu No. 58’ citrus fruit peel are illustrated in **Figure 3**. The contents of WSP, CSP, and TP increased with fruit development, while the pattern of change in NSP was less evident (**Figures 3A–D**). Overall, at 150 DAF, most Ca treatments showed higher WSP, CSP, NSP, and TP contents in the peel compared to Si treatments. Notably, the CSP and TP contents in C3 were notably the highest (1.49 mg/g DW and 6.91 mg/g DW), while those in S1 were notably the lowest (0.87 mg/g DW and 5.11 mg/g DW). Among the WSP, CK peel exhibited consistently higher levels, especially at 150 and 190 DAF, with significant levels of 3.38 mg/g DW and 8.35 mg/g DW, respectively. While the HC content from 110 to 190 DAF showed an increasing and then decreasing trend, the CEL content exhibited a decreasing trend (**Figures 3E–F**). The CEL content was significantly higher under the C3 treatment, whereas the HC content showed the opposite trend. The HC content in silicon-treated peel remained consistently higher, particularly under S4 and S5 at 150 DAF (434.94 mg/g DW and 603.69 mg/g DW).

### 3.4 Cell wall enzymes, antioxidant enzymes, and MDA

**Figure 4:**
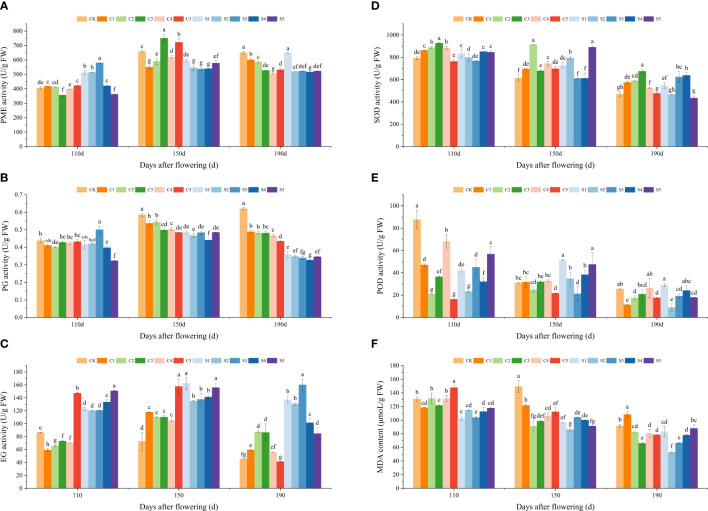
Effect of different treatments on cell wall hydrolase, antioxidant enzyme activity, and MDA content of the ‘Okitsu No.58’ citrus fruit peel. **(A)** PME, **(B)** PG, **(C)** EG, **(D)** SOD, **(E)** POD, **(F)** MDA. C, Ca treatment; S, Si treatment. The parameter values presented in each figure are indicated as mean ± standard error (n=3). Different letters denote statistically differences between different treatments (Tukey test, *P* < 0.05).

**Figures 4A–F** depict the variations in cell wall enzymes, antioxidant enzyme activities, and MDA contents of ‘Okitsu No. 58’ citrus fruit peel. PME, PG, and EG activities exhibited an increasing and then decreasing trend over time, while SOD, POD, and MDA displayed a decreasing trend. PME and PG activities were notably lower in Si-treated peel compared to Ca treatment, particularly under S5 at 110 DAF (361.73 U/g FW and 0.32 U/g FW). Conversely, EG exhibited a tendency of higher enzyme activity in Si-treated peel than in Ca treatment. Furthermore, PG activity in CK treatment was significantly higher at 150−190 DAF. EG activity was significantly lower at 110 DAF and notably higher at 150 DAF and 190 DAF for all Ca treatments compared to CK, except for C5. EG activity was notably higher in all Si-treated samples compared to CK.

SOD activity under all Ca treatments, except for C5, was significantly higher than that under CK during various periods, notably in C2 and C3. At 110 DAF, either Ca or Si treatments moderately reduced POD enrichment. Between 150 and 190 DAF, MDA content was significantly lower in all Ca treatments, excluding C1, than in CK, while all Si treatments exhibited lower MDA levels than CK throughout all periods. Additionally, MDA content under Si treatment was significantly lower than under Ca treatment, particularly notable in S2 at 190 DAF, where it reached 52.87 μmoL/g FW.

### 3.5 Fruit quality

**Figure 5:**
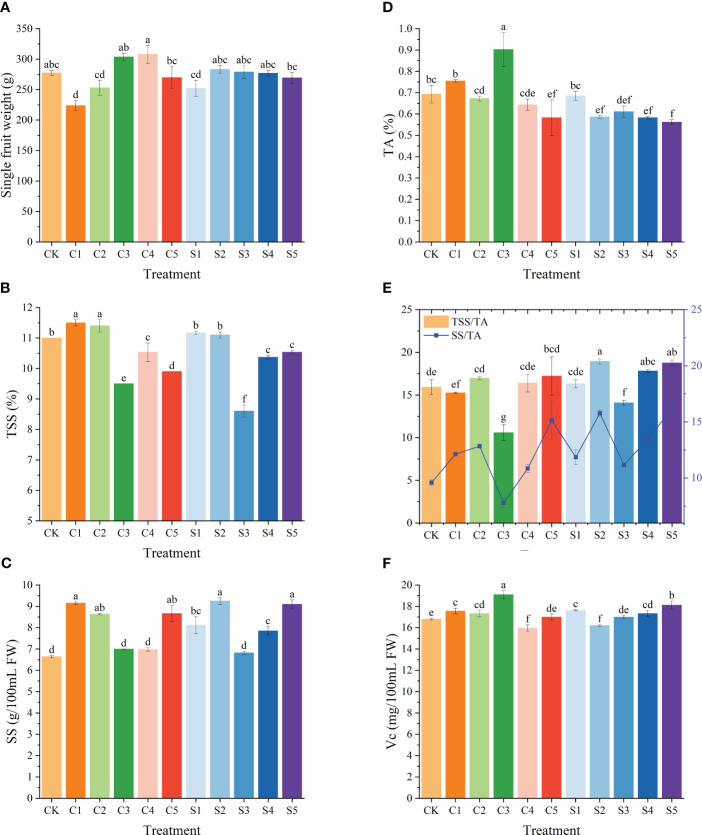
Effect of different treatments on the fruit quality of the ‘Okitsu No.58’ citrus. **(A)** Single fruit weight, **(B)** TSS, **(C)** SS, **(D)** TA, **(E)** TSS/TA and SS/TA, **(F)** V_C_. C, Ca treatment; S, Si treatment. The parameter values presented in each figure are indicated as mean ± standard error (n=3). Different letters denote statistically differences between different treatments (Tukey test, *P* < 0.05).

The impact of Ca or Si foliar fertilizers on the fruit quality of ‘Okitsu No. 58’ citrus is illustrated in **Figure 5**. In comparison to the CK, only the C1 treatment significantly decreased fruit weight per fruit, while other treatments did not exhibit notable differences. TSS were notably lower under C3 and S3 (9.5% and 8.6%, respectively) and significantly higher under C1, C2, S1, and S2. Further analysis of SS content revealed that C3, C4 and S3 also had substantially lower contents of 6.99%, 6.98% and 6.82%, respectively. Moreover, C3 demonstrated the lowest TSS/TA, and SS/TA ratios (10.59 and 7.79, respectively), yet displayed significantly higher TA at 0.90% and Vc at 19.09 mg/100mL. Overall, Si fertilizer treatments exhibited notably higher TSS/TA, and SS/TA ratios compared to those of Ca. This suggests that Si contributes to enhancing fruit quality in ‘Okitsu No. 58’ citrus.

### 3.6 PCA and correlation analysis

**Figure 6:**
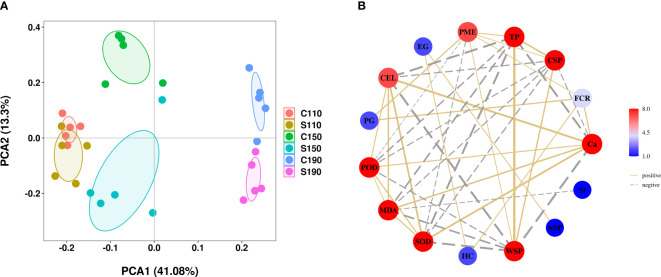
PCA and correlation analysis. **(A)** PCA score plot, **(B)** Relationship plot between indicators with significant differences in correlations. C110 and S110, Physiological indices of the ‘Okitsu No. 58’ peel at 110 DAF; C150 and S150, Physiological indices of the ‘Okitsu No. 58’ peel at 150 DAF; C190 and S190, Physiological indices of the ‘Okitsu No. 58’ peel at 190 DAF; FCR, fruit cracking rate.

PCA was conducted to elucidate the changes in peel composition of ‘Okitsu No. 58’ citrus fruit under different treatments and time points. The PCA results demonstrated a distinct separation of peel substances among the various treatment groups at 150 DAF (**Figure 6A**), suggesting that either Ca or Si treatments exerted differential effects on each physiological index of the peel, particularly evident at 150 DAF. Subsequent correlation analysis revealed a significant and positive correlation between fruit cracking rate and PME, PG, HC, and Ca, while the correlation with Si did not reach statistical significance. Moreover, Ca exhibited significant positive correlations with CEL, POD, MDA, and SOD, but significant negative correlations with WSP, TP, and CSP (**Figure 6B**). These findings underscore the significant regulatory impact of Ca on fruit cracking rate and various physiological indicators.

## 4 Discussion

Ca or Si reduce fruit cracking by increasing the resistance of the fruit to cracking (Li and Chen, 2017; Lü et al., 2021). In this study, we observed that either Ca or Si fertilizer treatments promoted Ca or Si accumulation in the peel, with particularly notable Ca enrichment under C3 and C4 peels (**Figure 2A**). Furthermore, when combined with fruit cracking rate, we found C3, with a higher Ca content in the peel, exhibited the lowest fruit cracking rate. This finding aligns with an earlier study on litchi (Huang et al., 2001). This may be attributed to the high uptake of exogenous Ca by the C3 peel, creating conditions to ionic cross−linking between cell wall polymers, especially between pectin molecule chains, which in turn reduces the fruit cracking rate (Davis, 1991). However, the reasons for the high rate of fruit cracking despite the fact that C2, C4 and C5 peel Ca content was also higher remain to be investigated. Under Si treatment, S4 at 150 DAF exhibited a lower fruit cracking rate (6.25%). It is speculated that this is because the treatment promoted the Ca uptake in the peel, consequently enhancing the peel’s resistance to cracking (**Figure 2A**). Similarly, in tomato studies, the addition of Si was found to significantly enhance Ca uptake, which the researchers consider to be a specific mechanism by which the plant copes with stress, but the mechanism of action remains to be explored (Zhang et al., 2023).

Fruit cracking is closely associated with the developmental stage of the peel, and the metabolism and structure of the peel’s cell walls play crucial roles in determining fruit cracking (Monselise et al., 1976; Jona et al., 1989; Huang et al., 2006; Li and Chen, 2017). In this study, although TP content in the peel showed an increasing trend, there was no noticeable difference between the CK and C3 treatments. Further research showed that WSP content under C3 was considerably lower than CK, while levels of CSP and NSP were notably higher (**Figures 3A–D**). Similar results were observed in pepper research (Liu et al., 2022). This can be explained by Ca inhibiting the increase of WSP, reducing the degree of protopectin degradation (CSP and NSP), stabilizing the cell wall structure, enhancing peel resistance to cracking, and significantly reducing the cracking rate (Huang et al., 1999). The lower activities of PME and PG under C3 would further contribute to its reduced WSP (**Figures 4A, B**), a relationship supported by correlation analysis (**Figure 6B**). However, Si primarily enhances crack resistance by reducing PME and PG enzyme activities and maintaining high CSP levels, particularly under the S4 treatment. Moreover, CEL and HC also play important roles in maintaining cell wall structure (Yang et al., 2011). Previous studies have indicated that litchi fruits prone to cracking exhibit reduced CEL and HC contents in their peel cell walls compared to crack−resistant varieties, suggesting that this discrepancy contributes significantly to the reduced mechanical strength of the peel in crack-prone varieties (Huang et al., 1999). In our study, C3 exhibited higher CEL but notably lower HC levels compared to the CK (**Figures 3E, F**), a trend consistent with the observed EG activity at 110 DAF (**Figure 4C**). However, the reason for the significantly higher EG activity of C3 compared to CK at 150 and 190 DAF requires further investigation. It is noteworthy that although the difference in CEL content between S4 and CK was not significant, the HC content was significantly higher. This disparity may also contribute to the reduced frequency of fruit cracking observed under S4. In addition, fruit cracking is also closely related to antioxidant enzyme activity (Zhang et al., 2021; Huang et al., 2024). In this study, SOD activity was higher under C3 treatment, especially at 110 and 190 DAF, being 16.93% and 43.99% higher than that of the CK, respectively. In contrast, POD activity was noticeably lower than CK. In addition, the C3 treatment had lower MDA levels. These findings indicate that C3 exhibited a stronger antioxidant capacity (Wang et al., 2013)and peel extensibility (Elstner, 1982; Campa, 1991), which could be significant factors contributing to its decreased fruit cracking rate. The Si results were similar to those Ca treatment.

**Figure 7:**
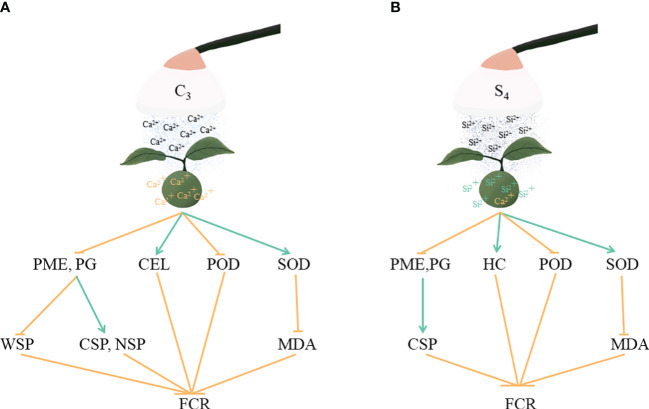
Diagram of the mechanism of action of C3 and S4 in controlling fruit cracking. **(A)** C3, **(B)** S4. FCR, fruit cracking rate. Orange arrows indicate inhibition and blue arrows indicate facilitation.

Previous studies have shown that spraying Ca or Si improves the intrinsic quality of fruit (Wang et al., 2022; Dou et al., 2023). Our results also showed that Ca or Si fertilizer treatments, except for the C3 treatment, had a beneficial effect on ‘Okitsu No. 58’ citrus fruit quality, mainly in terms of increasing the fruit SS/TA ratio (**Figures 5A–F**). Notably, treatment C3 resulted in the lowest TSS/TA and SS/TA (10.59 and 7.79), likely due to Ca fertilization inducing metabolic rearrangements in the fruit, consequently delaying ripening (Viviana Martins et al, 2021). In summary, Ca or Si fertilizers were effective on ‘Okitsu No. 58’ citrus fruit cracking, with C3 and S4 exhibiting relatively better in our results. The specific mechanisms of action are shown in **Figure 7**, both of which control fruit cracking by affecting the metabolism of cell wall substances and the antioxidant system of the fruit peel. However, further validation of our results is necessary through investigations involving other citrus cultivars.

## 5 Conclusions

The study’s findings revealed that the spraying of Ca or Si fertilizers reduced the fruit cracking rate of ‘Okitsu No. 58’ citrus compared to CK. Specifically, at 150 DAF, CK exhibited a fruit cracking rate of 18.29%, whereas C3 and S4 showed rates of only 2.47% and 6.25%, respectively. Both Ca treatment, S4 and S5 led to an increase in elemental Ca content in the peel. In addition, Ca or Si treatments may also affect WSP production by decreasing PG activity in the peel, and further respond to fruit cracking by increasing SOD activity, and decreasing MDA content in the peel. In fruit quality, most of the Ca or Si fertilizers helped to improve the conventional quality of the fruit. The combined analysis concluded that either Ca or Si foliar fertilizer application was beneficial in reducing fruit cracking in ‘Okitsu No. 58’ citrus with C3 (0.3 g/L of Ca) and S4 (0.4 g/L of Si) being relatively more effective.

## Data availability statement

The original contributions presented in the study are included in the article/supplementary material. Further inquiries can be directed to the corresponding authors.

## Author contributions

TW: Conceptualization, Data curation, Writing – original draft, Writing – review & editing. LT: Conceptualization, Validation, Writing – original draft, Writing – review & editing. ZC: Investigation, Writing – original draft. YTY: Investigation, Writing – original draft. YY: Investigation, Writing – original draft. ZZ: Data curation, Writing – original draft. LD: Data curation, Writing – original draft. MZ: Data curation, Writing – original draft. GS: Data curation, Writing – original draft. SH: Writing – original draft. JW: Writing – original draft. BX: Data curation, Writing – original draft. ZW: Funding acquisition, Project administration, Writing – original draft, Writing – review & editing.
